# Circulating miRNAs reflect early myocardial injury and recovery after heart transplantation

**DOI:** 10.1186/1749-8090-8-165

**Published:** 2013-07-01

**Authors:** Enshi Wang, Yu Nie, Qian Zhao, Wei Wang, Jie Huang, Zhongkai Liao, Hao Zhang, Shengshou Hu, Zhe Zheng

**Affiliations:** 1 State Key Laboratory of Cardiovascular Disease, Fuwai Hospital, National Center for Cardiovascular Diseases, Chinese Academy of Medical Sciences and Peking Union Medical College, Beijing, 100037, People’s Republic of China; 2 Department of Cardiac Surgery, Research Center for Cardiac Regenerative Medicine, Fuwai Hospital, Chinese Academy of Medical Sciences and Peking Union Medical College, Beijing, China; 3 Department of Heart Transplantation, Fuwai Hospital, Chinese Academy of Medical Sciences and Peking Union Medical College, Beijing, China; 4 Department of Cardiac Surgery, State Key Laboratory of Cardiovascular Disease, Fuwai Hospital, Chinese Academy of Medical Sciences, Xicheng District, Beijing 100037, China

**Keywords:** Circulating miRNAs, Myocardial injury, Ischemia-reperfusion injury, Heart surgery, Heart transplantation

## Abstract

**Background:**

MicroRNAs (miRNAs) are short, single-stranded and non-coding RNAs, freely circulating in human plasma and correlating with vary pathologies. In this study, we monitored early myocardial injury and recovery after heart transplantation by detecting levels of circulating muscle-specific miR-133a, miR-133b and miR-208a.

**Methods:**

7 consecutive patients underwent heart transplantation in Fuwai hospital and 14 healthy controls were included in our study. Peripheral vein blood was drawn from patients on the day just after transplantation (day 0), the 1^st^, 2^nd^, 3^rd^, 7^th^ and 14^th^ day after transplantation respectively. Serum from peripheral blood was obtained for cardiac troponin I (cTnI) measurement. Plasma was centrifuged from peripheral blood for measuring miR-133a, miR-133b and miR-208a by quantitative reverse transcription polymerase chain reaction (qRT-PCR). The plasma concentration of miRNAs were calculated by absolute quantification method. The sensitivity and specificity of circulating miRNAs were revealed by receiver operating characteristic curve (ROC) analysis. Correlations between miRNAs and cTnI / perioperative parameters were analyzed.

**Results:**

Similar to cTnI, miR-133a, miR-133b and miR-208a all showed dynamic changes from high to low levels early after operation. The Sensitivity and specificity of miRNAs were: miR-133a (85.7%,100%), miR-208a (100%,100%), and miR-133b (90%,100%). Correlations between miRNAs and cTnI were statistically significant (p < 0.05), especially for miR-133b (R^2^ = 0.813, p < 0.001). MiR-133b from Day 0-Day 2 (r > 0.98, p < 0.01), and cTnI from Day 1- Day 3 (r > 0.86, p < 0.05) had strong correlations with bypass time, particularly parallel bypass time. Obviously, miR-133b had a better correlation than cTnI. Circulating miR-133b correlated well with parameters of heart function such as central venous pressure (CVP), pulmonary capillary wedge pressure (PCWP), cardiac output (CO) and inotrope support, while cTnI only correlated with 3 of the 4 parameters mentioned above. MiR-133b also had strong correlations with ventilation time (r > 0.99, p < 0.001) and length of ICU stay (r > 0.92, p < 0.05), both of which reflected the recovery after operation. The correlation coefficients of miR-133b were also higher than that of cTnI.

**Conclusions:**

The dynamic change in circulating muscle-specific miRNAs, especially miR-133b can reflect early myocardial injury after heart transplantation. And miR-133b may have advantages over cTnI in forecasting graft dysfunction and recovery of patients after operation.

## Background

MicroRNAs (miRNAs) are single-stranded, non-coding RNAs with a length of 19–25 nucleotides, which negatively regulate gene expression by recognizing complementary messenger RNAs (mRNAs) and prohibiting their translation into functional protein [1]. The expression profile of miRNAs is found to be tissue-/cell-specific [2]. And aberrant expression of miRNAs can directly reflect disease status. Recently circulating cell-free miRNAs are found remarkably stable despite of repeated freeze-thawing, RNase digestion and other harsh conditions [3,4], partly because of the association with microvesicles or exosomes which are actively secreted or passively released into blood in physiological or pathological condition [5]; therefore, their potential as biomarker were evaluated in several cardiovascular diseases, such as coronary heart disease [6], acute myocardial infarction (AMI) [7-13], heart failure [14], and viral myocarditis [9]. MiR-133a, miR-133b and miR-208a, as cardiomyocyte-enriched miRNAs, become the common candidate miRNAs to study [7-13].

As the most effective therapy for end-stage heart disease, heart transplantation is widely used in many medical centers. The donor heart undergoes cold ischemia and ischemia-reperfusion injury during extracorporeal circulation, both of which induce necrosis and apoptosis of cardiomyocytes in donor heart and subsequent myocardial injury early after transplantation [15,16]. Review of the International Society for Heart and Lung Transplantation (ISHLT) Registry has shown that donor-related variables, particularly duration of myocardial ischemia, continue to emerge as an independent risk factor for early and late survival and cardiac allograft vasculopathy [17]. So it is very important to monitor the myocardial injury and give medical therapy at the early stage after transplantation. We hypothesized that the dynamic change of cardiomyocyte-enriched miRNAs in peripheral blood could reflect the myocardial injury and therefore predict recovery of heart function after heart transplantation. Thus we measured the circulating levels of miR-133a, miR-208a, and miR-133b in different time point after operation in the context of a sensitive myonecrosis marker, cardiac troponin I (cTnI), in 7 consecutive patients undergone heart transplantation.

## Methods

### Patient selection

From July 6^th^ to August 27^th^, 2011, 7 consecutive patients underwent heart transplantation in Fuwai Hospital (Beijing, China) were included in our study (Table 1). 14 healthy people were also included as a control group. The protocol of this study was carried out according to the principles of the Declaration of Helsinki and approved by the Medical Ethics Committee of Cardiovascular Institute and Fu Wai Hospital. Written informed consent was obtained from all the participants before enrolment.

**Table 1:** Patient characteristics

Number(n)	7
Sex(male)(n)	7
Age(years)	46.71 ± 12.31
Body weight(kg)	58.85 ± 9.35
Indication for cardiac transplantation(n)	
Coronary artery disease	1
Dilated cardiomyopathy	5
Restrictive cardiomyopathy	1
Perioperative parameters	
Cold ischemia time of donor heart(min)	284.29 ± 66.92
Bypass time(min)	189.86 ± 73.28
Aortic clamping time(min)	70.71 ± 9.14
CVP(mmHg)	5.92 ± 3.08
PCWP(mmHg)	9.16 ± 2.59
CO(L/min)	4.76 ± 1.00
Inotrope Score(micrograms /kg/min)	7.72 ± 3.48
Ventilation time(min)	38.43 ± 24.64
Length of ICU stay(days)	3.49 ± 2.46
Use of ECMO after transplantation(n)	2
Postoperative Echocardiography	
Left atrium diameter(mm)	41.29 ± 7.76
Left ventricular ejection fraction (%)	67.71 ± 7.27
Left ventricular end diastolic dimension (mm)	43.57 ± 4.47
Interventricular septum(mm)	9.43 ± 1.40
Right ventricular diameter(mm)	21.57 ± 5.88
Immunosuppressive therapy(n)	
Cyclosporine	6
Tacrolimus	1
Mycophenolate mofetil	7
Azathioprine	1
Sirolimus	0
Prednisone	7

All value are presented as numbers or mean ± SD. *ICU* Intensive care unit, *ECMO* extracorporeal membrane oxygenation, *CVP* central venous pressure, *PCWP* pulmonary capillary wedge pressure, *CO* cardiac output.

### Blood sampling from patients

6 ml venous blood samples were drawn from each patient immediately after transferred to ICU (day 0) and then at 6:00 am on the 1^st^, 2^nd^, 3^rd^, 7^th^ and 14^th^ day after transplantation respectively. For serum separation, 3 ml of each blood sample was put into a common test tube containing polyolefin resin (Greiner Bio-One, Frickenhausen, Germany) for at least 10 minutes at room temperature. Then the tube was centrifuged at 3000g for 20 minutes to obtain serum. The left 3 ml was put into a K_2_-EDTA-coated tubes (BD Biosciences, California, USA) and centrifuged at 1600 g for 10 minutes. The plasma was carefully transferred into a new RNA-free tube for second centrifugation at 16,000 g for 10 min to further eliminate cell debris. Then the plasma was aliquot into RNase-free tubes and stored at −80°C before RNA extraction.

### RNA extraction

Total RNA was extracted from 400 ul plasma, using the mirVana PARIS kit (Ambion, Warrington, United Kingdom) according to the modification of manufacturer’s instructions (as detailed in ref [3]), and subsequently eluted in 30 ul nuclease-free water. To date, several miRNAs such as miR-16 [18], or synthetic C. elegans miRNAs [3,6,19] have been established and validated to normalize for the miRNAs content in different body fluid. However, for detection of miRNA in plasma, synthetic C. elegans miRNAs were broadly used [3,6,19]. So in our experiment cel-miR-39, cel-miR-54, and cel-miR-238 were chosen and spiked in the plasma samples after combining the sample with 2 × Denaturing Solution (as a mixture of 25 fmol of each oligonucleotide in a 5 ul total volume) [3].

### Quantification of circulating miRNAs in plasma and cTnI in serum

5 ul RNA was put into 15 ul reverse transcribed (RT) reaction to generate cDNA using the TaqMan miRNAs Reverse Transcription Kit and miRNAs-specific stem-loop primers (Applied Biosystems, Foster City, USA). Quantitative reverse transcription polymerase chain reaction (qRT-PCR) was performed on ABI 7300 real-time PCR instrument (Applied Biosystems, Foster City, USA). The TaqMan 2× Universal PCR Master Mix (No AmpErase UNG) and TaqMan miRNAs Assay (Applied Biosystems, Foster City, USA) was used for quantification of miRNAs at 95°C for 10 min, followed by 95°C for 15 s (40 cycles) and 60°C for 1 min (40 cycles). The Ct value was defined as the cycle number at which the fluorescence exceeded the threshold. In our experiment the detection limit of Ct value was defined as 40. All PCR results were duplicated to evaluate the miRNAs’ expression level. cTnI levels in the serum were measured by the electrochemiluminescence method (Roche, Basel, Switzerland).

### Generation of standard curves for absolute quantification of miRNAs and normalization of experimental data

**Figure 1:**
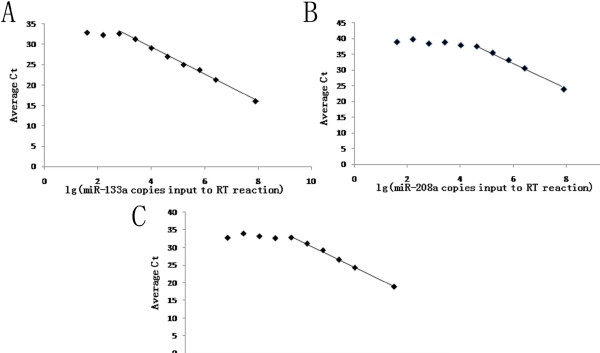
**Standard curves of miR-133a, miR-208a, and miR-133b for TaqMan qRT-PCR assays.** Standard curves were generated for each miRNAs assay using a dilution series of known input amounts of synthetic miRNAs oligonucleotide corresponding to the target of the assay (Table 1). Data points not on the trend line are those for which the measured Ct was interpreted to be below the linear range of the assay, and were not used for derivation of the trend line. **A**, Standard curve of miR-133a; **B**, Standard curve of miR-208a. **C**, Standard curve of miR-133b.

Synthetic single-stranded RNA oligonucleotides miR-133a, miR-208a, and miR-133b (miRBase Release v.19.0) were purchased from Shanghai GenePharma, China. Sequence information was provided in Table 2. Synthetic miRNAs were input into the RT reaction according to range of copies from reference [3,20]. The standard curves for miR133a, miR-208a, and miR-133b were plotted by Ct values versus copy number of the synthetic miRNAs as shown in Figure 1. Copies of endogenous miRNAs in plasma were then approximated according to their Ct values and the standard curve. A Normalization Factor was calculated by the Ct values of the three synthetic spiked-in C. elegans miRNAs [3,20]. Then the copies of a given miRNAs in each sample (calculated by the standard curves described earlier) were multiplied by the normalization factor corresponding to the sample to obtain a normalized copy number [3,20].

**Table 2:** Description of commercially purchased RNA oligonucleotides used in standard curve and spike-in experiments

**miRNA**	**Manufacturer of synthetic miRNA**	**Synthetic miRNA (oligo) sequence**
cel-miR-39	Shanghai GenePharma	UCACCGGGUGUAAAUCAGCUUG
cel-miR-54	Shanghai GenePharma	UACCCGUAAUCUUCAUAAUCCGAG
cel-miR-238	Shanghai GenePharma	UUUGUACUCCGAUGCCAUUCAGA
hsa-miR-133a	Shanghai GenePharma	UUUGGUCCCCUUCAACCAGCUG
hsa-miR-208a	Shanghai GenePharma	AUAAGACGAGCAAAAAGCUUGU
hsa-miR-133b	Shanghai GenePharma	UUUGGUCCCCUUCAACCAGCUA

### Cardiac function evaluation

Swan-Ganz Catheter (Edwards Lifesciences, California, USA) was implanted through external jugular vein of patients before operation. Hemodynamic parameters such as central venous pressure (CVP), pulmonary capillary wedge pressure (PCWP) and cardiac output (CO) were measured on 0 day, the 1^st^ day, 2^nd^ day, or 3^rd^ day postoperation (within 48–72 hous after catherization). A score adapted from Wernovsky and his colleagues was used to quantify inotrope use for our patients [21]. Inotrope score is a reliable index of postoperative cardiac function; higher scores indicate poorer cardiac function. The score was calculated by obtaining the total amount of inotropic support the patients received at each sampling point (on 0 day, the 1^st^ day, 2^nd^ day, and 3^rd^ day postoperation) and then entering the data into the equation as follows: Inotrope score = Dopamine + Dobutamine + ([Epinephrine + Norepinephrine + Phenylephrine] × 100) + Milrinone × 10. Units of inotrope dosage used in this equation were in micrograms per kilogram per minute.

### Statistical analyses

All statistical data were presented as mean ± standard deviation (SD) unless indicated otherwise. Pearson correlation analyses were used for miRNAs themselves or miRNAs versus cTnI and clinical parameters. Comparisons between 2 groups were performed with Student’s t-tests for Gaussian data or Mann–Whitney tests for non-Gaussian data. For comparisons of more than 2 groups, one-way ANOVA was used, followed by post hoc testing using Bonferroni correction for more groups. The sensitivity and specificity of each miRNAs was calculated by receiver operating characteristic curve (ROC) analyses. All p values were two-sided and p values <0.05 were considered statistically significant. All analyses were performed using SPSS version 17.0.0 (SPSS Inc, Chicago, USA).

## Results

### Levels of circulating miR-133a, miR-208a and miR-133b elevated in patients after heart transplantation

**Figure 2:**
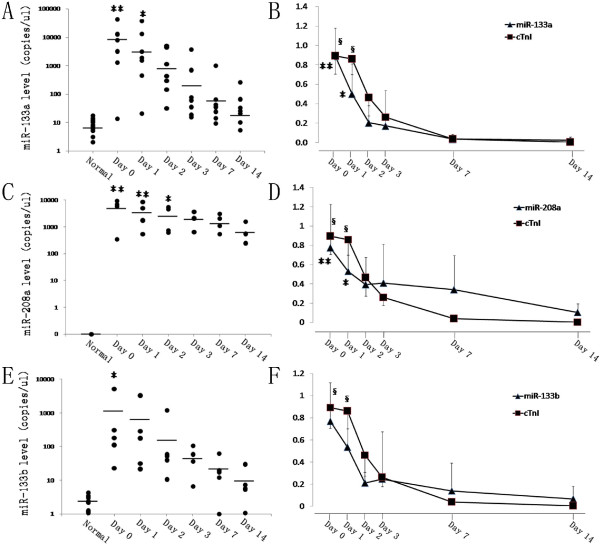
**Dynamic changes of miRNAs and cTnI in patients at different time points after heart transplantation.** Figure **A**, **C**, **E**, copy number of miR-133a, miR-208a, and miR-133b in plasma compared to healthy control from Day 0 to Day 14. Figure **B**, **D**, **F**, time course of miR-133a, miR-208a, miR-133b and cTnI in patients from Day 0 to Day 14. The data have been normalized to the peak level that each microRNA and troponin I achieved in each patient. **P < 0.01, *P < 0.05 vs healthy control in figure **A**, **C**, **E**; **P < 0.01, *P < 0.05 for miRNAs vs Day 14, §P < 0.01 for cTnI vs Day 14 in figure **B**, **D**, **F**.

The levels of circulating miR-133a, miR-208a, and miR-133b were elevated to peak just after the transplantation (Day 0) (Figure 2A, 2C and 2E). MiR-133a increased to 11675 ± 3481 copies/ul (p = 0.001), miR-208a to 5544 ± 1757 copies/ul (p < 0.001), while miR-133b to 1173 ± 227 copies/ul(p < 0.05) as compared to healthy controls. After that the expression levels of all three miRNAs in plasma declined gradually from Day 0 to Day 14. However, miR-133a on Day 1 was 8198 ± 1363 copies/ul, which was still higher (p < 0.05) than those in controls, so was miR-208a (3966 ± 1580 copies/ul, p = 0.001). There seemed to be no difference for miRNAs in patients compared to controls after 2 days since heart transplantation except miR-208a, which was still significant at Day 2 (2852 ± 550 copies/ul, p < 0.05). Whereas, miR-133a, miR-208a, and miR-133b all showed a trend to decline gradually from Day 0 to Day 14, which was similar to serum cTnI (Figure 2B, 2D and 2F). Fold changes of miR-133a, miR-208a and cTnI on Day 0 or Day 1 was significantly higher compared to Day 14 (p < 0.05), although there were no significance on other time points. Of the 3 miRNA in plasma, miR-133a was the most abundant on Day 0, then followed by miR-208a and miR-133b. In all samples, concentration of miR-133a and miR-208a was statistically higher than that of miR-133b (p < 0.05 and p = 0.001 respectively), while there was no difference between miR-133a and miR-208a. So we infer that circulating miR-133a and miR-208a are more enriched than miR-133b in plasma of patients after heart transplantation.

### Circulating miRNAs reflect myocardial injury effectively

**Figure 3:**
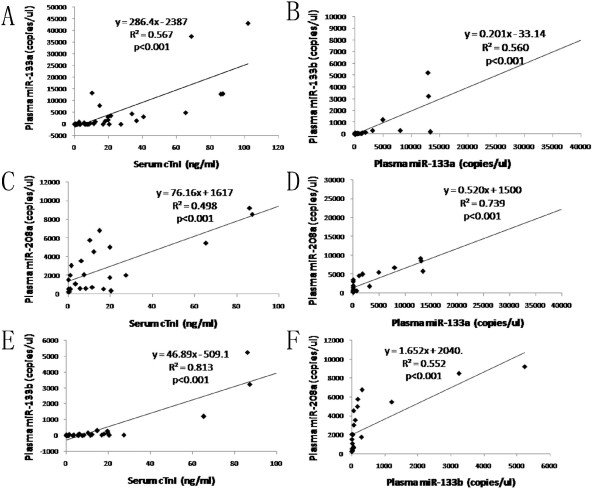
**Correlations of circulating miRNAs with cTnI and miRNAs themselves.** Figure **A**, **C**, **E**, circulating miR-133a, miR-208a, and miR-133b correlated with cTnI significantly (p < 0.001), especially miR-133b; Figure **B**, **D**, **F**, circulating miR-133a, miR-208a, miR-133b correlated with each other significantly (p < 0.001).

**Figure 4:**
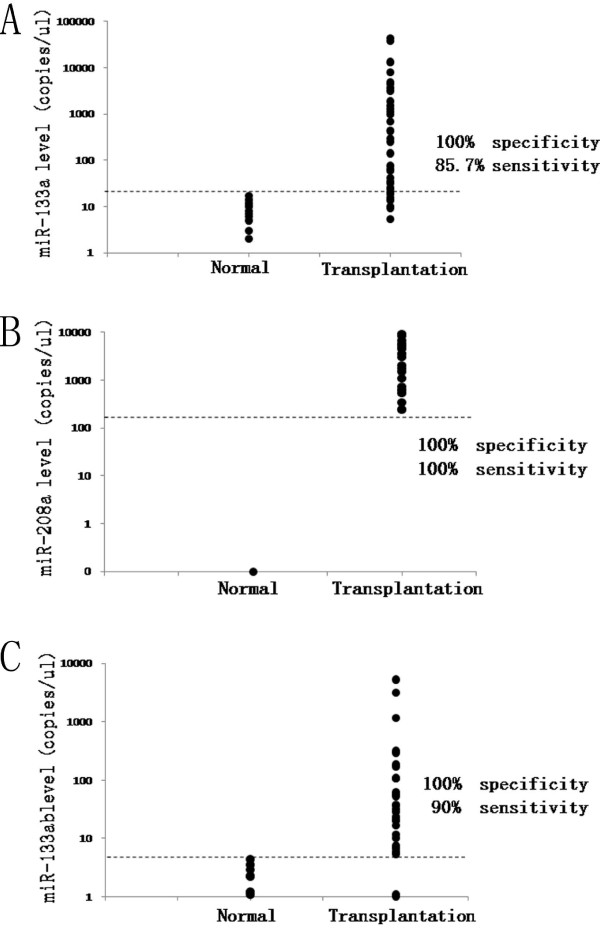
**Sensitivity and specificity of circulating miRNAs.** Figure **A**, Circulating miR-133a had 85.7% sensitivity and 100% specificity, whereas miR-208a had 100% sensitivity and specificity in figure **B**. In figure **C**, for miR-133b, the sensitivity is 90%, and specificity is 100%.

To evaluate whether circulating miRNAs can reflect the myocardial injury after heart transplantation, correlations between circulating miRNAs and cTnI were analyzed. We can see that all of these three circulating miRNAs in plasma correlated well with serum cTnI (p < 0.001) (Figure 3). MiR-133b had the stronger correlation with cTnI (R^2^ = 0.813, p < 0.001; Figure 3E) compared with the correlations between miR-133a/miR-208a and cTnI (R^2^ = 0.567, p < 0.001 and R^2^ = 0.498, p < 0.001 respectively; Figure 3A and 3C). For correlations among miRNAs themselves, miR-133a, miR-208a and miR-133b correlated strongly with each other (p < 0.001) (Figure 3B, 3D and 3F). However, miR-208a which was expressed exclusively in heart, had a 100% sensitivity and specificity (Figure 4B), but it couldn’t be detected in all patient samples (57.14%), and not existed in healthy controls at all. Although miR-133a was found in all samples and miR-133b could only be detected in 71.42% samples and 56.25% controls, miR-133b had a better sensitivity than miR-133a (90% sensitivity and 100% specificity vs. 85.71% sensitivity and 100% specificity; Figure 4A and 4C).

### Clinical correlations

To evaluate the reason for rising of circulating miRNAs after transplantation and whether these miRNAs could predict the recent prognosis of patients, correlations of cirulating miRNAs at different time points with perioperative parameters were analyzed (Table 3 and Table 4). Neither circulating miRNAs nor cTnI had relationships with cold ischemia time of donor heart. However, miR-133b from Day 0-Day 2, cTnI from Day 1- Day 3, and miR-133a on Day 2 did have strong correlations with bypass time. The correlation coefficients of both miR-133a and miR-133b were over 0.98, which is higher than cTnI (Table 3). It seemed that length of bypass time contribute to elevations of miR-133b, cTnI and miR-133a. Next we divided bypass time into 2 parts: the aortic clamping time and parallel bypass time when there was a low level of reperfusion despite of persistent ischemia of heart. Interestingly, only miR-133a on Day 3 negatively correlated with the aortic clamping time, and miR-133b had a stronger association with parallel bypass time than cTnI from Day 0 to Day 2 (Table 3).

**Table 3:** Correlations between circulating miRNA and perioperative parameters

		**Day 0**		**Day 1**		**Day 2**		**Day 3**	
		**p**	**r**	**p**	**r**	**p**	**r**	**p**	**r**
Cold ischemia time	miR-133a	0.355	0.415	0.251	0.502	0.139	0.618	0.49	0.316
	miR-208a	0.819	0.181	0.416	0.584	0.268	0.732	0.365	0.635
	miR-133b	0.413	0.48	0.433	0.463	0.405	0.487	0.796	0.161
	cTnI	0.137	0.621	0.14	0.617	0.247	0.505	0.137	0.62
Bypass time	miR-133a	0.607	0.238	0.337	0.663	**0.001**	**0.992**	0.073	0.721
	miR-208a	0.337	0.663	0.13	0.87	0.276	0.724	0.999	0.001
	miR-133b	**0.001**	**0.992**	**0.003**	**0.984**	**0.001**	**0.989**	0.801	−0.15
	cTnI	0.073	0.712	**0.012**	**0.866**	**0.001**	**0.962**	**0.009**	**0.881**
Aortic clamping time	miR-133a	0.139	−0.618	0.182	−0.57	0.889	−0.066	**0.036**	**−0.785**
	miR-208a	0.479	0.521	0.235	0.765	0.086	0.914	0.746	0.254
	miR-133b	0.397	0.495	0.437	0.458	0.415	0.479	0.455	−0.443
	cTnI	0.553	−0.273	0.927	−0.043	0.748	0.15	0.966	−0.02
Parallel bypass time	miR-133a	0.477	0.325	0.294	0.464	0.013	0.861	0.807	0.114
	miR-208a	0.358	0.642	0.164	0.836	0.33	0.67	0.978	−0.002
	miR-133b	**0.002**	**0.988**	**0.003**	**0.983**	**0.002**	**0.987**	0.854	−0.115
	cTnI	**0.043**	**0.769**	**0.006**	**0.898**	**<0.001**	**0.972**	**0.004**	**0.911**
Ventilation time	miR-133a	0.307	0.454	0.177	0.575	**0.002**	**0.934**	0.615	0.233
	miR-208a	0.317	0.683	0.12	0.88	0.266	0.734	0.977	−0.023
	miR-133b	**<0.001**	**0.999**	**<0.001**	**0.996**	**<0.001**	**0.999**	0.877	−0.097
	cTnI	**0.02**	**0.834**	**0.001**	**0.951**	**<0.001**	**0.997**	**<0.001**	**0.97**
Length of ICU stay	miR-133a	0.14	0.617	0.051	0.753	**0.002**	**0.932**	0.279	0.477
	miR-208a	0.366	0.634	0.156	0.844	0.321	0.668	0.989	0.011
	miR-133b	0.017	0.94	**0.024**	**0.926**	**0.02**	**0.934**	0.711	−0.029
	cTnI	**0.005**	**0.904**	**0.007**	**0.892**	**0.007**	**0.891**	**0.004**	**0.913**

Statistically significant correlations are depicted in bold.

**Table 4:** Correlations between circulating miRNA and parameters of cardiac function

	**CVP**		**PCWP**		**CO**		**Inotrope score**	
	**p**	**r**	**p**	**r**	**p**	**r**	**p**	**r**
miR-133a	**0.001**	**0.656**	0.719	0.098	0.450	−0.290	**0.001**	**0.605**
miR-208a	**0.011**	**0.677**	**<0.001**	**0.962**	0.401	−0.599	**0.018**	**0.581**
miR-133b	**0.002**	**0.692**	**0.017**	**0.730**	**0.049**	**−0.880**	**<0.001**	**0.861**
cTnI	**<0.001**	**0.728**	0.117	0.407	**0.043**	**−0.681**	**<0.001**	**0.756**

Statistically significant correlations are depicted in bold.

CVP, PCWP, CO, and inotrope score are indices of heart function. Higher CVP, PCWP, inotrope score and lower CO indicate worse dysfunction of heart. MiR-133a, miR-208a, miR-133b, and cTnI had positive correlations with CVP and inotrope support (Table 4). However, only miR-133b and miR-208a had positive correlations with PCWP; there were negative correlations between miR-133b/cTnI and CO (Table 4). So miR-133b was the only one that had the correlations with CVP, PCWP, CO, and inotrope score at the same time.

To evaluate recent prognosis, ventilation time and length of ICU stay were included. Ventilation time was strongly correlated with miR-133b from Day 0-Day 2, cTnI from Day 0-Day 3, and miR-133a on Day 2 (p < 0.05) (Table 3). The correlation coefficients of miR-133b were over 0.99, still the highest of all. MiR-133b from Day 1-Day 2, cTnI from Day 0-Day 3 showed correlationship with length of ICU stay (p < 0.05) (Table 3). The correlation coefficent of miR-133b at each time point was higher than that of cTnI.

## Discussion

Recent studies on circulating cardiomyocyte-enriched miRNAs as biomarker for cardiovascular disease are growing rapidly because of their rapid release kinetics, cardiac selectivity, and stability in plasma [7-14,22,23]. In coronary artery disease, miR-133a, miR-208a, and miR-133b are considered cardio- or skeletal muscle specific and released into peripheral blood after AMI [10,12,23]. As we know, donor hearts for heart transplantation underwent cold ischemia (organ-preserving cold storage interval) and extracorporeal circulation before the heart completely restored function in recipients. Necrosis and apoptosis of cardiomyocytes occurred after ischemia in donor hearts [15,16,24-26]. Exosomes or microvesicles containing miRNAs will be released from cardiomyocytes into blood [27]. So miRNAs, enriched in heart like miR-133a, miR-208a and miR-133b, will be detected in plasma. To our knowledge, it is the first report to use circulating miR-133a, miR-208a, and miR-133b monitoring donor heart injury after transplantation. Several new important observations are found in our study: 1) levels of circulating miR-133a, miR-208a and miR-133b are elevated in patients after heart transplantation and show a dynamic change; 2) circulating miR-133a, miR-208a, and miR-133b could reflect the heart injury after transplantation effectively; 3) circulating miRNAs are suggested to be a good predictive parameter for graft dysfunction and recent prognosis of patients after operation, especially miR-133b, which might be better than cTnI.

In our study, the levels of circulating miR-133a, miR-208a and miR-133b were peaked just after heart transplantation, which was similar to cTnI. The time point of Day 0 was at the moment patients just transferred into intensive care unit (ICU) from operating room after heart transplantation. So all of our sampling time points were after operation, not during the operation. Maybe that’s the reason why the cTnI and circulating miRNAs peaked at the same time point, not like reference before that circulating miRNAs peaked in 1–4 hours which was earlier than cTnI after AMI [10,13]. Despite of it, 3 of 7 patients did show a peak of cTnI at Day 1 later than miRNAs that leads to a general mild slope from Day 0 to Day 1. All of these three miRNAs showed sharp slopes (Figure 2), which was similar to dynamic change of miRNAs in ST-segment elevation myocardial infarction (STEMI) patients [10,28]. Overall of it, there was no difference between trend of cTnI and miRNAs (p > 0.05). Like cTnI, circulating miR-133a, miR-208a and miR-133b showed gradually declined trend as time passed, which was similar to the trend of implanted heart recovering from injury after cold ischemia and extracorporeal circulation insults, then restoring function as a normal heart.

Studies before showed circulating miRNAs correlated with cTnI or cardiac troponin T (cTnT) at varying degrees [9,19,29]. In our study, we found three circulating miRNAs were correlated with cTnI well, especially miR-133b, which was strongly associated with cTnI (R^2^ = 0.813, p < 0.001) (Figure 3E). Since cTnI is widely used as a myonecrosis marker for diagnosis of AMI [30], we would like to infer that circulating miRNAs could reflect the myocardial injury of donor heart after transplantation. Heart-specific miR-208a got a 100% sensitivity and specificity (Figure 4B), but it could only be detected in 57.14% samples. This result was similar to the report in which miR-208a was expressed at low levels in three of nine AMI patients and undetectable in plasma samples from healthy human [10]. The low detected rate limits its application in the future. MiR-133a could be measured in all patients, but its sensitivity is only 85.71% (Figure 4A), while miR-133b with a 90% sensitivity and 100% specificity seemed to be the compromise (Figure 4C).

As we know, extrocoporeal circualtion could lead to heart injury [26]. The present study showed bypass time was strongly associated with miR-133b on Day 0 (r = 0.992, p = 0.001), Day 1 (r = 0.984, p = 0.003), and Day 2 (r = 0.989, p = 0.001) (Table 3). This is better than cTnI with later time points and lower values. The parallel bypass time that stands for reperfusion period plays a major role in elevation of miR-133b, or in other words, heart injury. Experimental studies have indeed shown that reperfusion itself initiates a process by which part of the salvageable myocardium fails to be rescued [31,32]. Oxygen depletion during ischemia inhibits mitochondrial function, which leads to a surge of reactive oxygen species (ROS) during reperfusion [32,33]. ROS are toxic to the cell and could lead to cell death. Then more miR-133b were released from dead cardiomyocytes into plasma. That may explain the high correlations of miR-133b concentration with parallel bypass time. Although miR-133b is present in sekeletal muscle and bypass causes skeletal muscle ischemia and reperfusion, previous study confirmed circulating miR-133b didn’t elevate, but decreased within 24 h following acute ischaemia of skeletal muscle and then back to control level [10]. So Elevation of miR-133b from skeletal muscle sources could be excluded in our study. The present study suggests miR-133b play a better role than cTnI in reflecting myocardial reperfusion injury (Table 3).

It is well-known that elevated cTnI or cTnT from donor heart is associated well with donor heart dysfunction or graft failure on hemodynamic, echocardiography or inotrope support [34-37]. In our study, cTnI levels in recipients were correlated well with CVP, CO and inotrope support at different time points after heart transplantation (Table 4). The tendency was in accordance with previous studies [34-37], although the cTnI data in our experiment were from recipients after transplantation but not from donors. We also found these 3 circulating miRNA were associated with hemodynamic indices and inotrope support. Among them, miR-133b was still the best one to predict graft dysfunction, because miR-133b was the only one that had correlations with CVP, PCWP, CO, and inotrope support. The other 2 miRNAs and cTnI had correlations with only 2 or 3 of the 4 parameters (Table 4). Evidence from our experiment suggested that circulating miR-133b predict graft dysfunction better than cTnI.

MiRNAs like miR-133a, miR-208b can provide prognostic information in acute coronary syndrome [12,23]. In this study, we found miR-133b had the best correlation with ventilation time and length of ICU stay (Table 3). The length of ventilation and ICU stay reflects the recovery of patients after operation. So circulating miR-133b not only plays an important role in monitoring heart injury but also acts as a prognostic factor for patients’ recovery. Recent functional studies indicated that miR-133 had effects in the regulation of stress-induced myocyte survival with an antiapoptotic role [38]. This might point towards a cardio-protective role for these miRNAs in heart transplantation, which needs to be addressed thoroughly in future studies.

Some limitations are avaliable in the current study. First of all, the sample size is small. A large number of patients undergoing cardiac surgery will be included in our following study. Secondly, We didn’t collect the blood samples during the operation, which may partly explain the peaks of miRNAs were not earlier than that of cTnI. Thirdly, miRNA measurement is time-consuming at present, limiting the clinical use of the results. Technology needs to be developed to allow quick bedside test and broad use of miRNAs as biomarkers.

## Conclusions

The present study suggests circulating miRNAs, especially miR-133b be a new sensitive marker for monitoring and forcasting myocardial injury and recovery after heart transplantation. Our investigation also uncovers a new field for biomarker studies in cardiovascular surgery, such as coronary artery bypass grafting (CABG), cardiac valve replacement, or congenital heart disease (CHD) surgery, most of which undergo extracorporeal circulation.

## Abbreviations

cTnI: Cardiac troponin I; miRNAs: microRNAs; mRNAs: messenger RNAs; AMI: Acute myocardial infarction; qRT-PCR: quantitative reverse transcription polymerase chain reaction; SD: Standard deviation; ROC: Receiver operating characteristic curve; ROS: Reactive oxygen species; CABG: Coronary artery bypass surgery; CHD: Congenital heart disease; ISHLT: International Society for Heart and Lung Transplantation; STEMI: ST-segment elevation myocardial infarction; AMI: Acute myocardial infarction; CVP: Central venous pressure; PCWP: Pulmonary capillary wedge pressure; CO: Cardiac output.

## Competing interests

The authors declare that they have no competing interests.

## Authors’ contributions

EW, ZZ and SH carried out the conception and design. EW carried out the sample collection, experiment, data analysis, and interpretation. YN participated in experiment. ZL and JH participated in the sample collection. WW participated in the interpretation. EW carried out writing the article, SH, ZZ, and HZ participated in the critical revision of the article and the statistical analysis. QZ participated in data analysis and language correction. All authors read and approved the final manuscript.
